# Inferring the Driver’s Lane Change Intention through LiDAR-Based Environment Analysis Using Convolutional Neural Networks

**DOI:** 10.3390/s21020475

**Published:** 2021-01-11

**Authors:** Alberto Díaz-Álvarez, Miguel Clavijo, Felipe Jiménez, Francisco Serradilla

**Affiliations:** 1 Department of Artificial Intelligence, Escuela Técnica Superior de Ingeniería de Sistemas Informáticos, Universidad Politécnica de Madrid, 28031 Madrid, Spain; francisco.serradilla@upm.es; 2 University Institute for Automobile Research (INSIA—UPM), 28031 Madrid, Spain; miguel.clavijo@upm.es (M.C.); felipe.jimenez@upm.es (F.J.)

**Keywords:** Convolutional Neural Networks, Intelligent Transportation Systems, lane change, ADAS, driver’s behaviour, autonomous driving

## Abstract

Most of the tactic manoeuvres during driving require a certain understanding of the surrounding environment from which to devise our future behaviour. In this paper, a Convolutional Neural Network (CNN) approach is used to model the lane change behaviour to identify when a driver is going to perform this manoeuvre. To that end, a slightly modified CNN architecture adapted to both spatial (i.e., surrounding environment) and non-spatial (i.e., rest of variables such as relative speed to the front vehicle) input variables. Anticipating a driver’s lane change intention means it is possible to use this information as a new source of data in wide range of different scenarios. One example of such scenarios might be the decision making process support for human drivers through Advanced Driver Assistance Systems (ADAS) fed with the data of the surrounding cars in an inter-vehicular network. Another example might even be its use in autonomous vehicles by using the data of a specific driver profile to make automated driving more human-like. Several CNN architectures have been tested on a simulation environment to assess their performance. Results show that the selected architecture provides a higher degree of accuracy than random guessing (i.e., assigning a class randomly for each observation in the data set), and it can capture subtle differences in behaviour between different driving profiles.

## 1. Introduction

Identifying a driver’s lane change intention (i.e., “when” as opposed to lane change execution, which deals with “how”), is of special interest in the area of Intelligent Transportation Systems (ITS) from the safety and prevention standpoint. Modelling behaviour accurately has many advantages. For example, it allows us to forecast the future status of the modelled object which offers the opportunity to anticipate possible scenarios and therefore be accordingly prepared.

The possibility of modelling a driver’s lane change intention profile opens several possibilities for the development of advanced systems (both hardware and software) that make use of the data these models infer.

One example is the use of the inferred information in a Vehicle-to-Everything (V2X) network. If these models’ predictions attain enough confidence, the ego vehicle intentions can be predicted and then disseminated along an inter-vehicular network, in which ADAS devices would use that information to improve their outputs (e.g., to offer information in embedded devices or to take decisions before risk situations).

Another example is the implementation of such models in the decision-making module of an autonomous vehicle, providing it the means to behave in a less artificial (thus more human) way and improving its penetration in a mixed driving scenario (humans and autonomous vehicles). These models would be used in the on-board control unit together with other modules (e.g., lane change execution [1]) for the development of a complete autonomous decision-making module.

However, driver’s behaviour is complex, especially when it comes to the lateral manoeuvres. It involves an extensive number of different factors, ranging from the driver’s personal situation to the surrounding environment, relationships with other vehicles or route planning, among others. Thus, a classic top-down approach to behavioural modelling based on prior analysis does not seem very accurate due to the amount of variability in the input space.

Therefore, in this paper we describe a bottom-up strategy based on deep-learning techniques, specifically in the use of CNN for the environment analysis around the vehicle in order to extract the key characteristics that define the behaviour according to specific driver situations. Although these tools represent a common practice in several computer vision and robotics problems, their use for traffic simulation is not so widespread, nor the use of spatial variables (i.e., images) along with non-spatial ones (i.e., all other indicators) into one single model. With this approach we aim to approximate the behaviour of generic and specific driver profiles to identify the lane change intention in an urban traffic environment, as a previous step of the lane change execution process analysed in [1].

The rest of this paper is organised as follows. Section 2 provides a literature review on modelling the driver’s behaviour, focusing on those based on Computational Intelligence approaches for modelling the lane change behaviour. Section 3 presents the details for the proposed model pipeline. Section 4 and Section 5 detail the experiments carried out and the results achieved, respectively. A results interpretation are discussed in Section 6. Finally, the research contributions and future research lines are discussed in Section 7.

## 2. Literature Review

The pioneering driver models date back to the 1950’s, when the longitudinal behaviour of a vehicle in its lane was studied. We can consider the reference [2] as the first in the area to study and model a vehicle’s behaviour as a function of the preceding vehicle (known as car-following model).

However, it is not until the work of Gipps [3,4] that a combined model is presented for the car-following scenario (including the free-flow one) and the lane change. Many of the contributions after the Gipps model have been in line with new regimes derived from it (e.g., stop-and-go [5,6] or emergency [7]), the use of perceptual thresholds (i.e., the minimum value of a stimulus which triggers a perception) in input measures to result in a more human-like behaviour [8], or the introduction of new Computational Intelligence techniques such as those based on fuzzy controllers [9] or Artificial Neural Networks (ANNs) trained with both synthetic [10] and real data [11,12].

The Gipps model is interesting in the context of lane change for two main reasons. The first one is that it proposes that a lane change is motivated by different reasons, reducing them to two for its theoretical framework: mandatory (i.e., the vehicle must perform a lane change manoeuvre) and discretionary (i.e., the lane change manoeuvre may improve some driving situation, such as comfort). The second is the fact that the lane change model is interchangeable, in other words, it allows the incorporation of a different lane change behaviour to the original if certain criteria are respected. Both factors led to the development of more sophisticated and powerful lane change models to improve the model, such as differentiating the type of adjacent lanes (e.g., slow and fast lanes [13]) or the incorporation of communication with surrounding vehicles in decision-making [14].

One of the limitations to be emphasised is the sequential nature of the decisions of the original model, which causes a decision to invalidate multiple branches in the lane change decision tree, causing discretionary lane changes hardly to be selected. This limitation has been addressed with traditional techniques, generally based on approximate reasoning, such as probabilistic trees [15] or Hidden Markov Models [16]. However, the problem remains in the fact that, to improve the predictive capacity of these models it is necessary to extend the number of input variables, with the consequent increase in the relationships between them and, therefore, the complexity of the models.

Computational Intelligence (CI) techniques are ideal in these environments because, on one hand, they are very robust (i.e., they allow working with noisy, incomplete or erroneous data) and on the other, they are capable of efficiently representing relationships between input variables, especially in the case of ANNs. One of the first proposals applying the ANNs approach to driver’s lane change behaviour was made by Chong et. al. [17]. The schema, a fuzzy controller modelled almost like a Multilayer Perceptron (MLP), allowed modelling both longitudinal and lateral behaviour, but with a very short time horizon due to the election of variables (only in-car measures such as steering angle or speed). In fact, classical models and those based on purely in-vehicle variables generally suffer from this problem.

Some later works are based on similar ideas, but using more complex inputs, and leaving aside representation as rules (and losing the ability to explain) to gain in space exploration of more distant relationships and predictions in time [18,19]. Interestingly, much of the research focuses on the exploitation of the temporal nature of the inputs via Recurrent Neural Networks (RNNs) [20,21], and there are very few that exploit the relationship between objects in the environment and the behaviour to be modelled.

CNNs, on the other hand, are mostly used for the identification and tracking of objects [22] and dangerous behaviours such as drowsiness [23] or distraction [24]. However, there are works that make use of their capacity to extract spatial patterns to infer behaviours, ranging from specific ones such as lane change execution [1] to more general (and diverse) ones such as differentiating between eating, drinking and driving [25,26].

These CI techniques have been used quite successfully in the problem of lane changing. Some studies address precisely the problem of identifying the intent to change lanes, achieving and average intention inference accuracy above 90%, but they usually address the problem using simulators [27] or few examples in simple environments such as highways [28,29].

Therefore, there are several models that work with the longitudinal model in any environment, generally adjusting the speed, but there are almost no models that predict lane changes in urban environments. For this reason, it is expected that the spatial pattern capture potential of CNNs will allow modelling some of the motivations that lead to lane changes in drivers.

## 3. Driver Modelling

A model using CNNs is proposed and, for this objective, it is essential the definition of the proper input data. Then, this section is divided in two parts: data gathering and preprocessing, where we explain how we deal with the input data and its transformation into proper data suitable to feed the model, and model proposal where both the CNN model to be used in the experiment and its modifications to address space-time constraints will be presented.

### 3.1. Data Gathering and Preprocessing

The selection of the input data fulfils the condition that must be naturally acquired in near future vehicles, equipped with current sensors and some new ones that are being now introduced. These variables are summarised in Table 1, including the sensor or device that measures or contains the desired information: Camera (C), Controller Area Network (CAN) Bus (B), GPS and Digital Map (G), and LiDAR (L).

At this point, it must be highlighted that recorded data are measurable variables, but human drivers consider several subjective or non-measurable variables that have influence on the final decision, apart from random or erroneous decisions. Current systems cannot take them into account.

The Traffic Light Signals (TLS) status is divided in three variables, one for each possible status (i.e., green, amber, and red). A similar approach is followed by the Lane change variable, the output of our model, where each of the values correspond to one of the three possible reactions of the driver, i.e., left, right, and no change.

The Available driving distance is also separated into three variables, but unlike the previous ones, they are continuous and can coexist (i.e., values in previous ones are discrete and mutually independent). They represent how many metres we can drive before the current, immediate left and immediate right lanes are non-transitable (either because the lane ends or because the route does not allow us to continue along it).

The rest of the section describes how the variables listed in Table 1 were acquired and processed.

#### 3.1.1. Vehicular Environment Mining

The key input variable that will improve the model performance in comparison with previous ones is the environment. From it, the system can get a glimpse vehicle’s surroundings in a similar way a human driver does. In our case, the vehicular environment is captured with a LiDAR.

Using a LiDAR for capturing the surrounding environment has several advantages, such as the precision (e.g., by night they do not suffer from headlight glares or from poor lighting environments) or accuracy in distances (i.e., camera-based calculations imply computational power). The main problem here is that a CNN requires a fixed-size input volume, and the preprocessing generated by a LiDAR has as many points as obstacles the laser beams encounter in their path, so there can be no guarantee that the input will be fixed-sized and then, it cannot be included directly as an input value.

**Figure 1:**
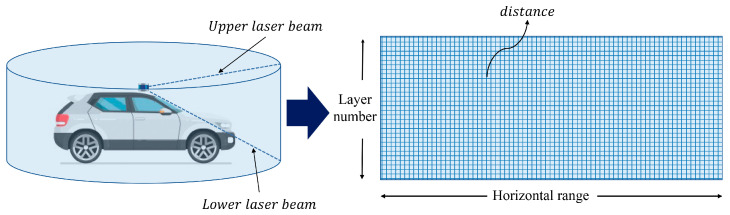
Outline of the depth map generation by means of the LiDAR layers’ recorded distances.

The approach adopted for this problem has been the transformation of the preprocessing into a depth map, that is, an image of a single channel where each “pixel” corresponds to the minimum distance detected by all the LiDAR beams crossing it. Figure 1 shows an example of such a representation. In our specific case, the generated depth map is 360 × 6 pixels, where 360 is the horizontal resolution and represents the total circumference in one-degree resolution, and 6 is the vertical resolution and represents the arc from −7 to 3 degrees (both included) in 2-degree resolution.

The selection of this vertical range is not arbitrary, but is due to both the technical characteristics of the device and its placement in the vehicle. From 5 degrees upwards, the laser beam hardly hits any obstacles. Similarly, from −9 degrees downwards, the laser directly hits the roof of the vehicle. Therefore, the portion of the image that really varies while driving is the one extracted from the specified interval.

#### 3.1.2. Avoiding Contextual Information Loss

The convolution layers operation in a CNN is based on scanning the image to be analysed with many filters (i.e., convolutions), extracting for each of them images where certain patterns of interest are highlighted. This is quite an issue for the depth map environment representation, since the left and right sides of the image (corresponding to the environment extracted directly from the front of the vehicle) are not connected, and thus no patterns can be identified.

Those filters traverse the images in two different ways depending on how they manage the edges: protruding the images (also known as *same* padding), in which case the part of the filter that does not touch the image is assumed to have an input of 0 and without protruding it (also known as *valid* padding). Unfortunately, none of these modes serve our purpose, as the front view of the vehicle is at the both ends of the image.

**Figure 2:**
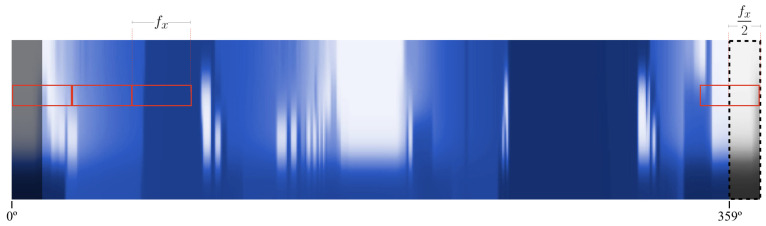
Dynamic padding scheme. Once the the left part of the depth map is appended to the right end of the image, the filter is able to cover the whole front of the vehicle, interpreting the possible patterns existing in it.

We approach this problem by using a padding scheme to a dynamically preprocessed image prior to feed the model, concatenating the left and right ends with the right and left ends respectively, with a width equal to half the size of the filter size of the first convolutional layer. Figure 2 shows the detail of this operation.

Being $f_{x}$ the filter width, the added section will be of $\frac{f_{x}}{2}$ width. The same procedure is done in order to add the right part of the depth map to the left, that is, adding the opposite region on both ends. This allows convolutions to identify patterns located at the back of the vehicle while navigating the left and right edges.

#### 3.1.3. Rest of Relevant Variables

Along with the vehicular environment, a set of additional variables has been recorded regarding the lane change intention detection process. Those were summarised in Table 1, along with the environment variable.

**Figure 3:**
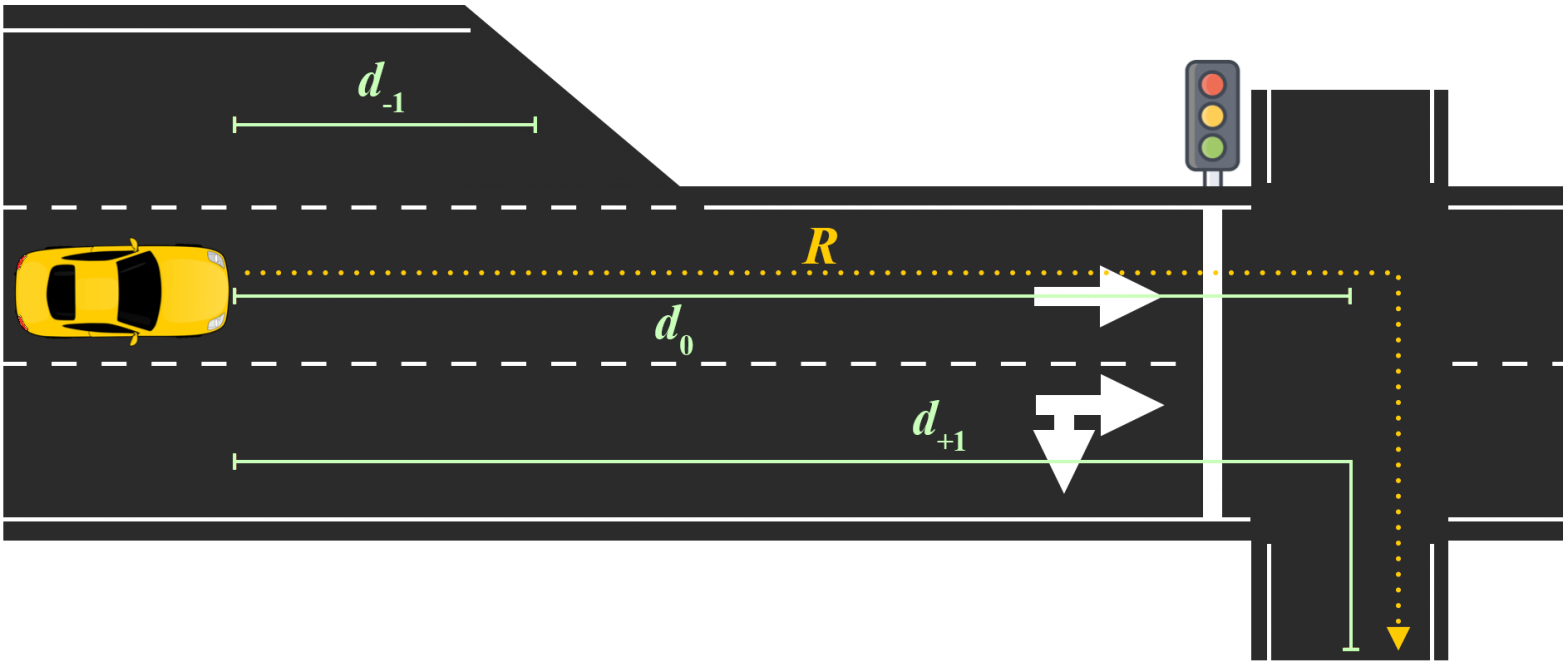
Example of how drivable distance works.

Although most of the variables are self-explanatory, the one referred to as Available driving distance one is perhaps a bit unusual. This variable has three values that describe, according to a pre-established route, how much can be driven in the left lane, the current lane, and the right lane. Figure 3 illustrates an example of how to understand this variable, where $d_{-1}$, $d_{0}$ and $d_{+1}$ are the distances that can be driven in the left, current and right lanes of the ego vehicle according to the route *R*. Once a lane change is performed, each variable will refer to different absolute lanes.

The values are extracted from the measurement of the distance at each instant from the position of the vehicle to the position of a series of checkpoints scattered throughout the scenario. These checkpoints refer to spatial positions of no return, i.e., those points from which it is impossible to continue driving along the pre-established route. For example, in Figure 3 it can be seen that, although there is no physical impediment to continue driving in the lane corresponding to distance $d_{0}$, the route imposes a right turn, so the distance $d_{0}$ is measured up to the point at which the change of direction can no longer be performed.

### 3.2. Model Proposal

Convolutional Neural Networks (CNN) [30] are feed-forward Neural Networks, but with a different topology than the well-known MLP. They are composed by two different components in their pipeline. Firstly, the input data are injected in a first component that we will denominate pattern extraction component, and its result will be introduced in an inference component, from which we will obtain the result.

The pattern extraction component is the key concept for a CNN. Instead of linear layers of neurons connected layer by layer as would be an MLP, the CNNs transform each input image into a set of new images resulting from the application of a certain operator, being the convolution operator the most important one (hence their name). The connections of these networks represent the convolution masks to be applied. Each layer of these networks defines a set of convolutions, and by stacking many of these layers and properly training the resulting network it is possible to solve several typical computer vision problems. In our case the problem to be solved is the lane change intention detection, for which an MLP is added as a final stage after the pattern extraction layers.

#### 3.2.1. Including Unrelated Non-Spatial Data

The data supplied to a CNN are represented as a *n*-dimensional volume, maintaining spatial consistency, that is, the closer the data are in the volume, the more related they are to each other. The problem here has to do with the fact that input data belong to two classes, which we will call *spatial* and *non-spatial*, where the former corresponds to the surrounding environment and the later corresponds to the rest of variables.

**Figure 4:**
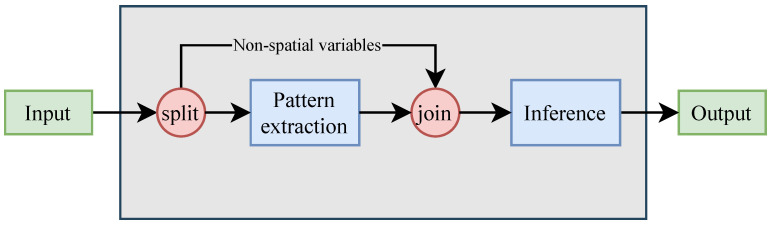
Non-spatial data are excluded from the Convolutional Neural Network (CNN)’s pattern extraction step and injected once the patterns have been inferred.

Since CNNs operate with spatially consistent data, it is not possible to merge spatial and non-spatial data at the input layer. To overcome this problem, considering that CNNs are comprised of the sequence of two components, feature extraction and inference, it has been decided to incorporate non-spatial data directly into the inference component, as shown in the Figure 4.

Prior to the aggregation, both the spatial data (the patterns extracted by the convolutions output) and the non-spatial data are normalized and included into the inference layer, avoiding the problem of incorporating them into the input layer along with all spatial  information.

#### 3.2.2. Avoiding the Temporal Dimension

CNNs are feed-forward networks, thus they cannot retain temporal information (e.g., speed or acceleration of the surrounding objects in the environment). For this purpose, based on [1], the datasets are transformed in such a way each sample contains not only the information for the current moment, but also moments before.

In particular, we will expand the model input to $X=X_{t}\cup X_{t-10}\cup X_{t-20}$ where *X*, $X_{t-10}$ and $X_{t-20}$ are the values for the variables in an instant *t*, t−1 seconds and t−2 seconds, respectively.

## 4. Experiments Presentation

A total of 13 drivers have driven on two different urban circuits with similar characteristics to obtain their driving profile data. The drivers are male and aged from 30 to 35 years. The drivers have been clustered into 3 groups according to their driving profiles, as suggested in [31]. Each profile, hereinafter referred to as $S_{1}$, $S_{2}$ and $S_{3}$ (corresponding to high-aggressive, low-aggressive and in-between respectively), shall be composed of the data collected from 5, 3 and 3 drivers respectively, while the remaining 2 were not clearly characterized and therefore were discarded.

The vehicle involved in data acquisition has been a Mitsubishi iMiEV equipped with a LiDAR placed at the top of the vehicle at 1.75 m high, a Microsoft Kinect Camera located behind the interior rear-view mirror of the vehicle, oriented to offer a front view, a GPS receiver equipped with differential correction and fixed in the same position of the LiDAR, and CAN Bus reader connected to the vehicle’s Bus. All the data has been synchronized at 10 Hz.

The two urban tracks, $R_{1}$ and $R_{2}$, used to acquire the driver’s data, are comprised of sectors with one, two and three lanes, TLS, crossings and their maximum speeds range between 30 kmh${}^{-1}$ and 50 kmh${}^{-1}$. $R_{1}$ has an estimated travel time of 30 min and will be used as a data source for model training; $R_{2}$, on the other hand, has an estimated travel time of 15 min, and will be used as a data source for testing the chosen model.

A driver model has been developed with the data collected from all drivers, regardless of which profile they belong to, called general model or $S_{A}$. It has been trained and tested with the data collected from $R_{1}$ and $R_{2}$ respectively, to check which architecture (if any) can extract the human lane change behaviour.

Once checked that the general model seems to capture real driver characteristics, the same network architecture has been trained and validated on the three specific datasets ($S_{1}$, $S_{2}$ and $S_{3}$, one for each driving profile) to check if the model can also capture their differentiating characteristics.

Finally, the trained models (both the general and the specific ones) are implemented within a simulation environment to check if the trained models are really capable of producing behaviours that could be considered as performed by the real profiles

### 4.1. Model Training

The final datasets for both training and testing are depicted in Table 2. In this table we can see the size of the three datasets (one for each different driving profile) and the whole dataset resulting from the union of those three. All datasets consist on 8653 inputs (i.e., each frame *t*, t−1 s and t−2 s) and 3 outputs for each sample (i.e., right, none, and left).

Training and test datasets are obtained from routes $R_{1}$ and $R_{2}$ respectively, and then preprocessed to obtain the datasets that we will call originals (those containing temporal information). Also, the values in the training columns are the number of original samples, without applying artificial augmentation.

**Figure 5:**
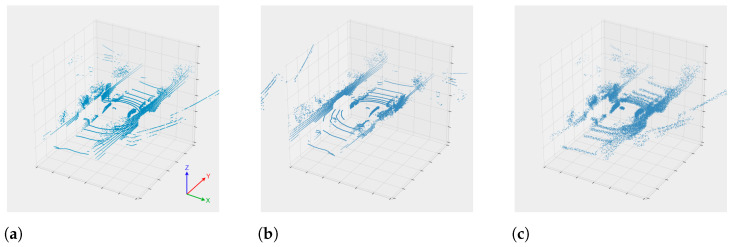
Example of the augmentation techniques: (**a**) The original pointcloud. (**b**) After a mirroring process. (**c**) After a shaking process (2 cm).

While the original test set is the one used in the testing task, the training set is dynamically modified during the training process in an effort to increase the overall generalisation of the models being inferred. For each epoch, the training test is divided randomly into two subsets, one for the actual training task (80%) and other for the validation task (20%). Then, during the epoch, the training set is dynamically increased by applying the mirroring (inverting the *Y* axis with respect of the XZ plane for each example) and shaking (each point for every original and mirrored example is shifted ±2 cm) tasks, thus quadrupling the number of available. Figure 5 illustrates two examples of this augmentation. The process of deriving the modified data is described in [1].

### 4.2. General Driver Profile

The training process has been carried out following the regularization technique known as early stopping, that is, stop training as soon as the validation error reaches a minimum. In our case, the number of epochs has been approximately $3\times 10^{4}$ epochs for all the trained models. The training process has been carried out with a batch size of 256 samples due to the use of GPU memory by both the size of the model and the size of the input during the training process.

Dropout [32] has been used as a regularization technique, so the rate of disconnections becomes one more meta-parameter to adjust. The activation functions are Rectified Linear Units (ReLU) in the convolutions and in the inference component, so the Glorot Initialization [33] will be used to initialize the network weights, as it is particularly designed for networks with this type of activation function.

As a gradient descent method, Adam [34] has been used along with an adaptive learning rate, which is no more than a smooth decrease in the learning rate to a minimum value throughout the training, so, in the early stages of training, the rate is high enough to converge quickly to areas of interest, but, at the same time, as training progresses, problems of bouncing around minima are avoided. It will be determined by the Equation (1):

(1) $$\alpha_{i}=\alpha_{min}+\left(\alpha_{max}-\alpha_{min}\right)\cdot e^{\frac{i}{d}}.$$

where $\alpha_{i}$ is the learning rate to apply in the epoch *i*, $\alpha_{min}$ and $\alpha_{max}$ the lower and upper rates that take the learning rate and *d* the decrease rate.

The models have been trained with values $10^{-5}$ and $10^{-1}$ for $\alpha_{max}$ and $\alpha_{max}$ respectively, and with a *d* parameter of $10^{-5}$. This values have been obtained after different trial and error tests.

### 4.3. Specific Driver Profile

The neural network architecture deduced for the general driver model has been used to train each of the different driving profiles, resulting in the so-called specific driving models. This training has been realized with the training data obtained from route $R_{1}$ for each driving profile.

Then, each of the models has been tested against the test data of each of the profiles, hoping that the model of each profile will better predict the test data set of their own profile rather than the others.

### 4.4. Simulation Environment

The Simulation of Urban MObility (SUMO) [35] simulator has been used to test how the models behave in a simulation environment. The simulator itself implements an Application Programming Interface (API) named Traffic Control Interface (TraCI) and offers a reference implementation in the form of a Python library also named TraCI.

TraCI allows the user to take over the simulation, enabling access to a large part of the information existing in each of the simulation steps (e.g., the state of a specific traffic light in an instant *t*), as well as executing commands that modify the behaviour of the elements (e.g., commanding a driver to execute a lane change).

A thin layer on top of the TraCI library has been developed in order to offer some newer functionalities such as virtual devices (i.e., LiDAR, GPS and CANBus) and high level operators (e.g., check_available_driving_distance and check_next_tls_status among others).

**Figure 6:**
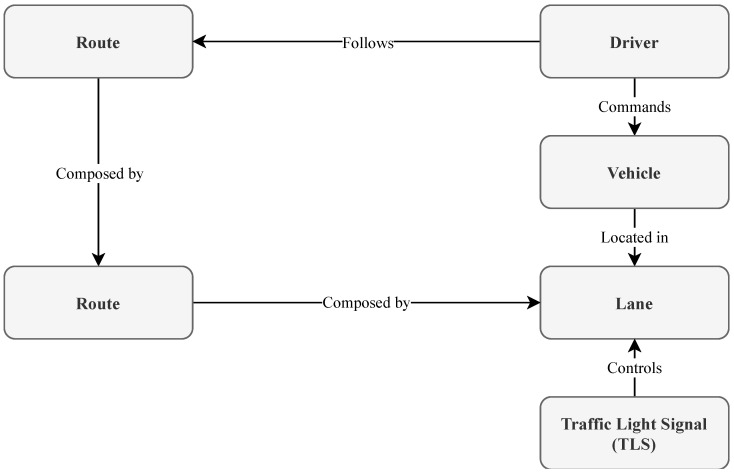
Outrun library main classes and how they are related.

A thin layer on top of the TraCI (we have called it outrun) have been implemented, which offers a perspective from the intelligent agents’ point of view. Figure 6 depicts a schematic of the main classes and their relationships.

In SUMO the concept of “vehicle” comprises both the physical vehicle attributes and the behaviour of the driver who controls it (known as Driver-Vehicle Object or DVO). We separate those concepts into the Vehicle (physical attributes of the vehicle) and Driver (the actual behaviour). The Driver class object must also include the Route description to be followed, which consists of an ordered list of Edges (to keep SUMO’s original nomenclature), formed by one or more Lane instances controlled by Traffic Lights Systems (TLS).

Each time we want to run a simulation with one or more DVOs following an specific behaviour, one or more instances of the Driver class must be created together with the corresponding behaviour models. Then, the framework will execute that behaviour at each simulation time step. It implements the trained models with the real data inside the agent, and for each step is fed with synthetic data extracted directly from the simulation, causing (or not) a lane change order that is directly the agent’s own action over the environment.

The SUMO simulation environment allows us to collect practically all the information proposed in Table 1 apart from the environment. However, since we can access to the position of the surrounding vehicles of the ego-vehicle, a simulated LiDAR have implemented that offers a preprocessing of similar characteristics to those captured by the original one. It will perform the following steps every tenth of a second:

1. Acquisition of every object (i.e., vehicle) position located at a radius *r* of the centre of the LiDAR.
2. Transformation of those objects into prisms with the dimensions specified by their properties (i.e., a prism with the same width, length and height as the actual vehicle of the experiment).
3. Computation of the preprocessing colliding such prisms.

In the simulated vehicle, the default SUMO lane change model has been replaced with each of the trained models ($S_{A}$, $S_{1}$, $S_{2}$ and $S_{3}$) for each one of the tests, maintaining the default longitudinal model provided by the simulator. The default values are described in the Table 3.

Both positive and negative acceleration have been obtained by computing the average on the real dataset examples. The maximum speed is the one attained in the whole dataset, whereas the width, length and height of the ego vehicle are the dimensions of the real car. Imperfection parameter applies a stochastic behaviour to the car, and the value is the recommended in [36].

Finally, the same route as the one used for the test circuit has been designed in the simulation environment, maintaining the vehicle flow measured during the test runs. Each proposed lane change model will be contrasted with the general lane change SUMO model [37] using the same values in the longitudinal model.

## 5. Results

### 5.1. Model Choice

The architectures of the best performing models are described in Table 4. The dropout rate used in the training of these models was 0.1, value obtained after a trial and error process. The name of the architectures is coded as follows: cF-*W*-*H* is a convolutional layer composed of *F* filters of width *W* and height *H*, whereas dN corresponds to a dense layer of *N* neurons.

The $CNN_{2}$ model will be used since it is not only the architecture with a higher accuracy while training, but also with the closest validation-training accuracy. Table 5 shows the confusion matrix for both the immediate 0.5 s and the 2.5 s window prediction using the test set.

It can be observed that immediate predictions reveal a clear bias towards no lane change and the model hardly infer when the driver tries to change lane. However, slightly increasing the time window size (+2 s) displaces many of these changes misclassified as None towards their respective real values (Left and Right) exceeding the random choice.

This makes sense since lane changes occur over several seconds. It should also be mentioned that in the evaluation of misclassified cases, some of the wrong classifications occurred between correct Left-Right classifications (e.g., Left-Left-Right-Left during a left lane change), suggesting that the model could be significantly improved by providing feedback on its previous output.

A relatively large network was needed to exceed the limit imposed by the random classification that is, hit a third of the cases by random guessing. This contrasts with the results obtained in [1], where classification values were much higher. The reason for this data disparity may have to do with the fact that the lane change decision operation is more complex to model than the lane change execution operation, where the decision is already made and we only have to deal how is going to be performed.

Furthermore, it was found that as the depth of the network increased, the over-fitting became more noticeable. Increasing the degree of regularization, while slightly decreasing the over-fitting, also decreased the overall accuracy notoriously. Therefore, it has been preferred not to use any pooling technique, keeping a smaller amount of convolution layers making the use of pooling layers unnecessary, as proposed in [38].

#### Specific Driver Profile

After selecting the $CNN_{2}$ architecture, the specific models are generated for each individual driver profile separately in order to verify whether the model is capable not only of capturing the properties of a lane change intention, but also the intrinsic differences between the different driving profiles.

Table 6 presents the accuracy achieved by the models of each of the profiles.

It can be observed that the achieved accuracy attained in the specific models is greater than the one achieved by the global model. It makes sense since the general model has been trained with data from all the profiles and therefore does not exactly obey the behaviour of any profile, whereas in the specific models it does.

In order to check the differentiation capability of the profiles with the selected architecture, the success rates in their respective test sets are compared. Table 7 shows the accuracy for each of the subjects compared to the rest

It can be seen that, when predicting a test set of a profile, the model trained with the training set of that profile has a higher accuracy than those trained with the training data of the other profiles. It can therefore be understood that the trained model is quite capable of extrapolating specific characteristics of a particular profile.

### 5.2. Validation of the Simulated Environment

Finally, the new model behaviour has been compared to the default lane change model included in SUMO [37] with its default values. The models’ inference times are in the order of milliseconds, so they could be executed in a real time system and our simulation environment. Still, our simulation environment has been executed with a 0.1 s time step to match the real time step with which the model has been trained.

The number of lane changes during the route have been considered as a model assessment indicator, since the route in the simulation environment has been designed in such a way that it reproduces the real test route in terms of distances, route structure and traffic flows. Because the model is exclusively oriented to lane change intention, other variables of driver behaviour that influence traffic flow are not taken into account such as car following modes, stop-and-go, etc.

A summary of the indicators extracted after running the simulations against the general model is shown in Table 8. It presents the real number of lane changes performed (mean and standard deviation of all drivers in the experiment) the number of lane changes performed by the SUMO model and the proposed one. All the simulation results are the mean μ and standard deviation σ of the resulting values after 10 different runs of the scenario.

Based on the results, the amount of lane changes in the SUMO model is significantly higher, and that of the simulation models slightly lower. The results shown in Table 9, Table 10 and Table 11 corresponds to the specific models, and are similar to those observed in the general model.

Taking into consideration the values resulting from simulation tests, it can be appreciated that the SUMO default model provides quite different results from the real values, while those of the simulated models are closer. However, in the latter case, the values are slightly lower than expected. We consider that there are two main factors for this behaviour: firstly, the simulation environment, which offers very little information about the environment so the depth map is much simpler than a real one; secondly, the low proportion of lane changes in relation to the total number of samples (this is very evident in the case of right-hand lane changes) biases the model towards not performing lanes changes, despite having trained the model in batches that preserve the output class ratio.

## 6. Discussion

The results offered by the model are reasonably good in terms of accuracy when compared to a mere random guessing.

Considering the accuracy achieved, and in the absence of lane change intention models in urban environments to compare with in the literature, it can be stated that according to the proposed inputs that this CNNs based model can predict behaviours with greater accuracy than random guessing. In fact, increasing the prediction window also increases predictive capacity for a general driving model.

Probably with another series of variables it may be possible to increase these model’s accuracy, but in this experiment, we are limited to the set of variables observable in real and simulation environments. Even with these limitations, the model provides reasonable results considering the complexity of urban environments and the variables that could have influence in a lane change decision, most of them impossible to obtain in a quantitative way such as driver’s mood or personal circumstances.

Also, the use of a lane change as a measurement of change intention affects to that computed accuracy. Though it is true that a lane change is caused by an intention, the opposite is not necessarily true. There are many situations in which the driver wishes to change lanes but, as it is impossible, he/she does not do so and therefore it is not measured.

It must be kept in mind that the CNN does not have the same “knowledge” as the human driver because of the sensing capacities and this fact reduces the ability of reproducing the reality. Furthermore, human drivers can anticipate situations based on experience or simple expectations but without using any measurable variable.

In addition, the accuracy values retrieved after training the specific driving profiles suggest that differentiating characteristics have been learned from these profiles. However, more work seems to be needed along these lines, as in view of the results, it appears that the input variables are not enough to produce a prediction with a success rate high enough to suggest that the model “mimics a human”.

## 7. Conclusions

In this paper, a driver model capable of predicting lane changes has been presented. The model overcomes the limitations of other models included in the literature which are based on simulations or are valid only for specific and simple scenarios and faces a very complex situation such as urban driving. The model is based on CNN and considers variables of the vehicle and its surroundings perceived using a LiDAR and postprocessed accordingly to obtain the relevant information to feed the network avoiding complex computational tasks of obstacles and other road elements identification and classification.

Although this identification is not carried out, traffic density can be estimated in an indirect way so the networks works with this relevant information for decision making. Considering the accuracy achieved, and in the absence in the literature of lane change intention models in urban environments, it can be stated that, according to the proposed inputs that this CNNs based model can predict, behaviours with greater accuracy than random guessing. In fact, increasing the prediction window also increases predictive capacity for a general driving model.

In order to improve the model, two main refinements are proposed for future developments. The first one is conducting more driving tests. The number of samples resulting from the number of drivers is rather low compared to the variability of the input space defined by the input variables. With a larger number of drivers, more delimited profiles and more quality data could be obtained, allowing the incorporation of more knowledge into larger networks, even avoiding the use of artificial data augmentation.

The second one is the identification of new variables. In this experiment variables have been limited to those that are easily extractable from both a real and a simulation environment. Identifying and extracting new variables from the environment could help to identify more decisive factors at the time of decision making by a given driver or driving profile.

In conclusion, even the registered limitations, the incorporation of these models reflect the behaviour of human driving profiles, predictable with some degree of inaccuracy, thus can be used for predicting lane changes in real driving conditions. This information could also be used to propagate in an inter-vehicular communication network to anticipate possible actions and events, and to prevent risk situations. It also can be used for supporting a better decision making process in autonomous vehicles that behaves in a more human-like manner or as lane choice model inside microscopic traffic simulation model DVOs.

## Figures and Tables

**Table 1:** Variables for the driver’s models.

Variable	Source	Units	Description
Environment	L	m	Surrounding environment as a pointcloud.
Speed	B, G	ms${}^{-1}$	Ego vehicle speed.
Distance from leader	C, L	m	Distance from the preceding vehicle.
Speed to leader	C, L	ms${}^{-1}$	Rel. speed with respect to the leader.
Distance from TLS	C, G	m	Distance from the upcoming TLS.
TLS status	C, G	{green, amber, red}	Status of the upcoming TLS.
Available driving distance	C, G	m	Distance that can be covered driving.
Lane change	C, L	{left, none, right}	Action performed (model output).

**Table 2:** Summary of the different datasets size.

Profile	Total			Left Change			Right Change		
	Training	Validation	Test	Training	Validation	Test	Training	Validation	Test
$S_{A}$	210,364	52,589	147,562	16,270	4087	11,299	7714	1927	5427
$S_{1}$	9598	23,989	66,527	8208	2046	5674	2332	583	1616
$S_{2}$	57,510	14,377	41,720	2833	700	2032	2674	667	1940
$S_{3}$	56,896	14,223	39,315	5299	1341	3593	2708	677	1871

**Table 3:** Values used in the simulation for the longitudinal models.

Profile	Value			
	$S_{A}$	$S_{1}$	$S_{2}$	$S_{3}$
Positive acceleration (ms${}^{-2}$)	2.7	3.1	2.6	2.2
Negative acceleration (ms${}^{-2}$)	3.5	4.6	3.8	2.1
Maximum speed (ms${}^{-1}$)	13			
Imperfection (∈[0,1])	0.5			
Width (m)	1.475			
Length (m)	3.395			
Height (m)	1.600			

**Table 4:** Architectures for the best performing models.

		Accuracy		
Architecture		Training	Validation	Test
$CNN_{1}$	c16-4-18 d64	0.512	0.486	0.424
**$CNN_{2}$**	**c16-4-18 d128**	**0.588**	**0.576**	**0.573**
$CNN_{3}$	c16-4-18 d128-d54	0.571	0.506	0.499
$CNN_{4}$	c32-5-36 C256-3-5-d256-d128-d16	0.463	0.339	0.321
$CNN_{5}$	c64-5-36 C256-3-5-d256-d128-d16	0.506	0.531	0.537
$CNN_{6}$	c16-4-18 C32-3-18-c64-2-18-d128	0.561	0.544	0.551
$CNN_{7}$	c16-4-18 C32-3-18-C64-2-18 d128-d64	0.601	0.298	0.297

Accuracy values for the best architecture are bolded.

**Table 5:** Confusion matrices for test set predictions.

(a) Immediate 0.5 s				
	Predicted			
		Left	None	Right
Real	Left	32.44%	47.11%	20.45%
	None	5.76%	60.50%	33.74%
	Right	12.31%	70.30%	17.39%
(b) 2.5 s window				
	Predicted			
		Left	None	Right
Real	Left	**42.86%**	**36.69%**	20.45%
	None	5.76%	60.50%	33.74%
	Right	12.31%	**45.13%**	**42.56%**

Bolded values show shifting towards good classification as prediction window is increased.

**Table 6:** Summary of achieved accuracy values for the general and the specific lane change models.

Profile	Accuracy		
	Training	Validation	Test
$S_{A}$	0.588	0.576	0.573
$S_{1}$	0.805	0.763	0.768
$S_{2}$	0.683	0.708	0.706
$S_{3}$	0.727	0.706	0.710

**Table 7:** Accuracy achieved by the profile specific models against their own and the other two test datasets.

	$S_{1}$	$S_{2}$	$S_{3}$
Model $S_{1}$	**0.768**	0.314	0.601
Model $S_{2}$	0.601	**0.706**	0.511
Model $S_{3}$	0.648	0.666	**0.710**

Bolded values show a better accuracy of the models with their respective test routes.

**Table 8:** Comparison between lane change models: real, Simulation of Urban MObility (SUMO) and the proposed general model.

	Real		SUMO		$S_{A}$	
	μ	σ	μ	σ	μ	σ
Left change	6	2.049	11.9	0.876	3.9	1.969
Right change	3.273	1.849	c12	0.667	1.2	0.919

**Table 9:** Comparison between lane change models: real, SUMO and the proposed model for profile $S_{1}$.

	Real		SUMO		$S_{1}$	
	μ	σ	μ	σ	μ	σ
Left change	6.4	2.408	11	1.414	2.8	1.476
Right change	2	2	10.7	1.703	2.3	1.418

**Table 10:** Comparison between lane change models: real, SUMO and the proposed model for profile $S_{2}$.

	Real		SUMO		$S_{2}$	
	μ	σ	μ	σ	μ	σ
Left change	4.333	1.528	11.4	1.776	1	0.6
Right change	4.333	1.155	11	1.333	0.6	0.483

**Table 11:** Comparison between lane change models: real, SUMO and the proposed model for profile $S_{3}$.

	Real		SUMO		$S_{3}$	
	μ	σ	μ	σ	μ	σ
Left change	7	1	10.5	0.707	3.8	1.932
Right change	4.333	0.577	11	0.943	1.93	0.919
